# The Effect of Zinc Supplementation on Lipid Profiles in Patients with Type 2 Diabetes Mellitus: A Systematic Review and Dose–Response Meta-Analysis of Randomized Clinical Trials

**DOI:** 10.1016/j.advnut.2023.08.006

**Published:** 2023-08-19

**Authors:** Mohammad Heidari Seyedmahalleh, Mohsen Montazer, Soraiya Ebrahimpour-Koujan, Leila Azadbakht

**Affiliations:** 1 Department of Community Nutrition, School of Nutritional Sciences and Dietetics, Tehran University of Medical Sciences, Tehran, Iran; 2 Department of Clinical Nutrition, School of Nutritional Sciences and Dietetics, Tehran University of Medical Sciences, Tehran, Iran; 3 Autoimmune Bullous Disease Research Center, Razi Hospital, Tehran University of Medical Sciences, Tehran, Iran; 4 Diabetes Research Center, Endocrinology and Metabolism Clinical Sciences Institute, Tehran University of Medical Sciences, Tehran, Iran; 5 Department of Community Nutrition, School of Nutrition and Food Science, Isfahan University of Medical Sciences, Isfahan, Iran

**Keywords:** zinc, lipid profile, type-2 diabetes mellitus, meta-analysis

## Abstract

Research on the effects of zinc supplementation on lipid profiles in people with type 2 diabetes mellitus (T2DM) has been inconsistent. This systematic review and meta-analysis was performed to summarize the current data on the effects of zinc supplementation on lipid profiles in patients with T2DM. Three online databases including PubMed, Scopus, and Web of Science were searched to find relevant studies published until September 2022. The exposure was zinc supplementation, and the outcomes were low-density lipoprotein (LDL), high-density lipoprotein (HDL), triglyceride (TG), and total cholesterol (TC). Fourteen randomized clinical trials consisting of 1067 patients were included in the statistical analysis. Significant improvement was observed in all 4 lipid profile components. Following zinc supplementation, a significant decrease was observed in TC (weighted mean difference [WMD]: −16.16; 95% confidence interval [CI]: −26.43, −5.89; *P* = 0.002), LDL (WMD: −6.18; 95% CI: −9.35, −3.02; *P* < 0.001), and TG (WMD: −13.08; 95% CI: −21.83, −4.34; *P* = 0.003). After analyzing 13 studies reporting HDL, a significant increase was seen (WMD: 3.76; 95% CI: 1.30, 6.22; *P* = 0.003). In a nonlinear dose–response analysis, a significant inverse association was observed between <12 wk zinc supplementation and TC, LDL, and TG (TC: WMD: −5, *P*_nonlinearity_ < 0.001; LDL: WMD: −5, *P*_nonlinearity_ = 0.07, TG: WMD: −16.5, *P*_nonlinearity_ = 0.006). Nonlinear dose–response analysis shows that the optimum elemental zinc dosage for the best response to the supplementation for TC, LDL, and TG are 120, 100, and 140 mg/d, respectively (TC: WMD: −5, *P*_nonlinearity_ < 0.001; LDL: WMD: −10, *P*_nonlinearity_ = 0.006, TG: WMD: −50, *P*_nonlinearity_ = 0.031). In conclusion, we found significant changes in all 4 components of the lipid profile through zinc supplementation in T2DM patients. Based on our findings, zinc supplementation may have profound favorable consequences on the lipid profile of T2DM patients, especially in the zinc-deficient group.

**Statement of Significance**

To the best of our knowledge, this is the first dose-response study on the effect of zinc supplementation on lipid profile in patients with diabetes, in addition to introducing new studies and addressing the shortcomings of previous studies. The findings of our study are clinically important and can be useful for medical and nutritional recommendations in patients with diabetes.

## Introduction

Type 2 diabetes mellitus (T2DM) is a growing worldwide health issue. At first, it was known as a constituent of metabolic syndrome and is commonly manifested by high amounts of blood glucose and insulin resistance [1]. Some subsequent comorbidities are correlated with T2DM, including hypertension, hyperlipidemia, renal dysfunctions, and other related organ failures [2]. Individuals with T2DM are highly risky candidates for both micro- and macrovascular disorders [3]. Ninety percent of all diabetics are assumed to be patients with T2DM [4], which, according to the latest reports, accounts for >6% of the global population. As the ninth cause of threats against life expectancy, T2DM resulted in 1 million deaths in 2017 [5]. Diabetes has a wide range of economic costs per capita from $242 in a low-income country like Mexico to $11,914 in the United States as a high-income country [6].

Etiology studies have suggested a combination of genetic and environmental factors. Environmental factors consist of aging, obesity, inadequate energy intake, alcohol consumption, and smoking. Visceral fat obesity is correlated with lower muscle mass and induction of insulin resistance [7]. Extra triglyceride (TG) stored in adipocytes makes them less sensitive to insulin and its suppression action on hormone-sensitive lipase and as a result, increases lipolysis and supply of free fatty acids (FFAs) into the circulation. FFA uptake by the liver and muscles competes with glucose and thereby reduces the metabolic utilization of blood sugar [8]. An excess of FFAs and blood glucose results in β-cell dysfunction through the progressing endoplasmic reticulum stress by stimulation of the apoptotic unfolded protein response mechanisms [9]. The lipid profile is influenced by the accumulation of FFAs. Betatrophin, serum triacylglycerol, and total cholesterol (TC) concentrations are significantly associated with T2DM [10]. A significant and positive correlation between oxidative stress status parameters and HDL cholesterol concentrations is found in patients with diabetes [11]. Indeed, metabolic and oxidative stress is the main cause of hormonal malfunction in patients with T2DM, and on the other hand, persistent hyperlipidemia gradually shifts an insulin response to the degree of insulin insensitivity [12]. Thus, the body’s antioxidative systems mediated by antioxidant micronutrients have an important role in detoxifying free radicals produced by lipid peroxidation [13]. Also, loss of some nutrients as a result of oxidative stress is observed in patients with T2DM, such as increased urinary zinc loss [14] or an obvious decrease in erythrocytes zinc concentrations [15]. As well as the aforementioned routes, zinc can be replaced with stress-inductive ions, such as copper and iron and lead to fewer lipid peroxide products [16]. Although studies have shown improvements in total oxidative stress levels by zinc supplementation through a reduction in lipid peroxidation [17] or a decrease in liver malondialdehyde concentration as a stress marker [18], on the other side some studies found no positive outcome neither in the markers of oxidative destruction and hydroxyeicosatetraenoic acid products concentrations [19] nor in glycemic control indices [20].

Overall, there is a need for a comprehensive and updated meta-analysis summarizing all eligible findings in this area. Therefore, the current meta-analysis was conducted to summarize current evidence to determine the effects of zinc supplementation on lipid profile indices in patients with T2DM. There is a noticeable privilege in our study and that is we are implementing a dose-duration response analysis of zinc supplementation on lipid profile in T2DM patients.

## Methods

This study was performed according to PRISMA [21].

### Search strategy

We implemented a comprehensive literature search of the online databases PubMed, Scopus, Web of Science, and Google Scholar up to September 2022. We did not limit our search strategy to any language or publication date restriction. All randomized, controlled, human trials that investigated the impact of zinc supplementation on lipid profile component concentrations in patients with T2DM were searched. In addition, the reference list of the relevant articles was reviewed not to miss any eligible trials. All searched studies were imported to the Endnote (X8) software for screening and removing duplicate references. In order to conduct a systematic search, the following keywords were considered as 3 concept queries: zinc OR “zinc supplement” OR “zinc sulfate” OR “zinc elemental” OR “Zinc Isotopes” OR “zinc gluconate” OR “Zinc Oxide” OR “Zinc Sulfate Heptahydrate,” and “lipid profile” OR Cholesterol OR “total Cholesterol” OR “TC” OR “VLDL” OR “VLDL Cholesterol” OR “Pre-beta-Lipoprotein Cholesterol” OR “Very Low Density Lipoprotein Cholesterol” OR “beta-Lipoprotein Cholesterol” OR “beta-Lipoprotein” OR “LDL Cholesterol” OR “Low Density Lipoprotein Cholesterol” OR “Low-Density Lipoproteins” OR “LDL” OR “HDL Cholesterol” OR “HDL Lipoproteins” OR “High-Density Lipoprotein” OR “alpha-Lipoproteins” OR “HDL” OR “High Density Lipoprotein Cholesterol” OR “HDL2 Cholesterol” OR “HDL3 Cholesterol” OR “alpha-Lipoprotein Cholesterol” OR Triacylglycerol OR Triglyceride OR “TG” and “Type 2 Diabetes Mellitus” OR “Type 2 Diabetes” OR “Diabetes Mellitus, Noninsulin-Dependent” OR “Diabetes Mellitus, Type II” OR “insulin resistant Diabetes” OR “glycemic control” OR “Maturity Onset Diabetes.”

### Inclusion criteria

We included eligible studies that met the following criteria: *1*) randomized controlled clinical trials (RCTs), *2*) studies conducted on adult subjects (≥18 y) with T2DM (no other types of diabetes), *3*) administered zinc in different chemical forms, including elemental, sulfate, gluconate, or any other isomers, *4*) RCTs with ≥1 wk duration of intervention, and *5*) controlled trials that reported mean changes and their SDs of lipid profile components (TG, TC, LDL, and HDL) throughout the trial for both intervention and control groups or provided information from which we could calculate effect sizes.

### Exclusion criteria

In this meta-analysis, we excluded animal studies, those with cohort, cross-sectional, and case–control designs, review articles, and ecologic studies. Trials without a control group whether placebo or other mixture designs, and those which were not randomized and/or were performed on children or adolescents without T2DM or patients affected by other diseases were excluded. Studies that did not define their design that we could not clarify whether they were blinded or randomized were also excluded.

### Data extraction

Two independent investigators extracted the required data from each eligible trial. The following information was extracted: name of the first author, publication year and country, individuals’ characteristics (mean age and sex), study design, sample size (control and intervention groups), type of zinc administered, the dosage and duration of supplementation, mean changes and their SDs of lipid profile throughout the trial for the intervention and control groups with their assessing units, the confounding variables adjusted in the analysis, and the data needed for quality assessment for risk of bias. We applied milligram per deciliter (mg/dL) as the most frequent unit for lipid profile components, and if a study reported in different units, we converted them to mg/dL.

### Quality assessment

We applied the Cochrane quality assessment tool for assessing the risk of bias for each study included in the current meta-analysis [22]. This tool contains 7 domains, including random sequence generation, allocation concealment, reporting bias, performance bias, detection bias, attribution bias, and other sources of bias. Each domain was given a “high risk” score if the study comprised methodological defects that may have affected its findings, a “low risk” score if there was no defect for that domain, and an “unclear risk"” score if the information was not sufficiently clear to detect the impact. The overall risk of bias for an RCT was considered: *1*) good; if all domains had “low risk,” *2*) fair; if 1 or 2 domains had “unclear risk,” and *3*) poor; if one or more domains had “high risk” or >3 domains had “unclear risk.” This process was implemented by 2 independent reviewers.

### Statistical analysis

Mean changes and their SDs between the baseline and endpoint of each of the 4 lipid profiles in the intervention and control groups were used to calculate the overall effect sizes. When mean changes were not reported, we converted them by considering changes in lipid concentrations during the intervention. We also transformed SEs, 95% confidence intervals (CIs), and interquartile ranges to SDs using the method of Hozo et al. [23]. In case the outcome was reported in millimoles per liter (mmol/L), we changed each 4-component unit to mg/dL by multiplying each one by a unique relevant ratio. The same happened for converting all trials’ dosage intake to elemental zinc if they reported the zinc sulfate or zinc gluconate dosage intake to have a comprehensive variable unit. To obtain the overall effect sizes, we applied a random-effects model that takes between-study variations into account. Heterogeneity was determined by the I^2^ statistic and Cochrane’s Q test. I^2^ value of >50% or *P* value of <0.05 for the Q-test was considered significant between-study heterogeneity [24]. To find possible sources of heterogeneity, subgroup analysis was conducted according to the predefined variables, including study design (randomized, randomized blinded, or parallel randomized blinded), sex (male, female, both), country (developed compared with developing), age (≤60 y compared with >60 y), participants’ health condition (with or without microalbuminuria) intervention type (elemental, sulfate, or gluconate, baseline serum concentrations of zinc), control type (placebo compared with nonplacebo), duration of the intervention (≥8 compared with <8 wk), matching for confounding variables (matched compared with not matched), adjustment for baseline levels of the outcome variable (adjusted compared with nonadjusted), baseline serum zinc (deficient, sufficient, and not reported), and study quality (good, fair, and poor). To determine the nonlinear effects of zinc dosage (mg/d) on lipid profile, fractional polynomial modeling was applied. Dose–response analysis was done considering duration and study population. Sensitivity analysis was used to detect the dependency of the overall effect size on a particular study. The possibility of publication bias was examined by the formal test of Begg adjusted rank correlation test beside Egger test and funnel plot. The meta-analysis was carried out using Stata, version 14.0 (Stata Corporation). *P* value of <0.05 was considered a significant level. Data extraction was performed using Microsoft Excel 2013, and data were imported from Excel into Stata 14.0 (Stata Corporation). All the statistical analyses, including meta-analysis, were conducted using the special commands for clinical trials in Stata 14.0. *P* value of <0.05 was considered as the level of statistical significance.

## Results

### Findings from the systematic review

#### Study selection

**FIGURE 1:**
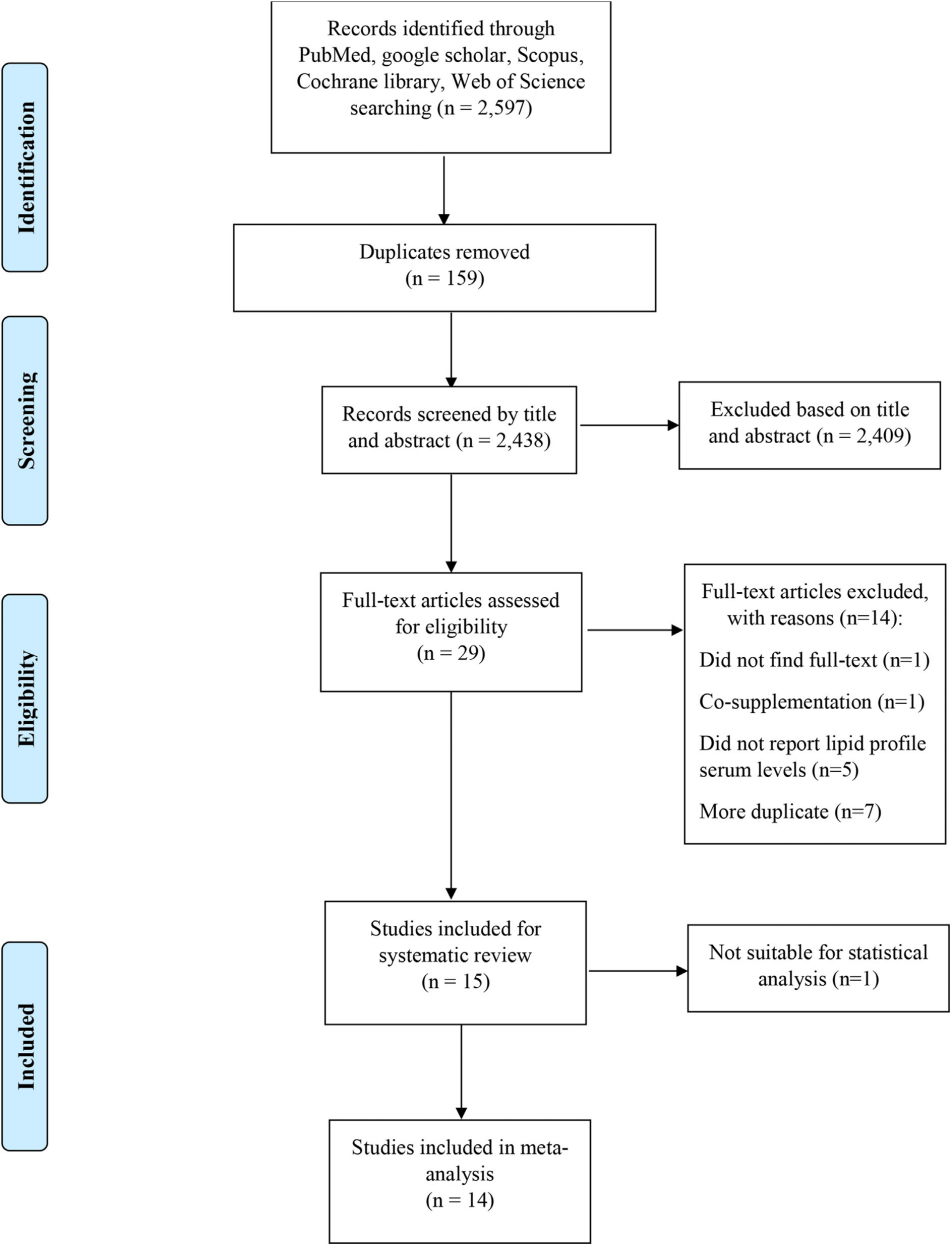
Study selection of the systematic search on the effect of zinc supplementation on lipid profile in patients with type 2 diabetes mellitus according to the PRISMA checklist.

We found 2597 articles in the preliminary electronic database search, of which 159 were duplicates. After removing duplicate articles, 2438 unique studies were screened, of which 2409 articles were excluded according to irrelevant titles and abstracts. The remaining 29 articles were reviewed. In the full-text step, 1 article’s full text could not be found, 1 article had a cosupplementation design, and 5 articles did not report lipid profile serum concentrations. Moreover, 7 more duplicate articles were excluded. Finally, 15 eligible studies were included [19,[25], [26], [27], [28], [29], [30], [31], [32], [33], [34], [35], [36], [37]]. Among all, 1 study was not suitable for statistical analysis. All the steps above are done according to the PRISMA checklist (Figure 1).

#### Study characteristics

The main features of the 15 studies [19,[25], [26], [27], [28], [29], [30], [31], [32], [33], [34], [35], [36], [37]] that examined the effects of oral intake of zinc supplements on the lipid profile components of patients with T2DM are shown in Table 1. In total, 1067 participants were included in the meta-analysis. All studies were published between 2006 and 2021. In terms of the country where the studies were conducted; 6 studies were in Iran [25,26,[32], [33], [34],^37^], and 1 study each in Australia [29], Iraq [38], Mexico [35], Singapore [19], Sri Lanka [30], Saudi Arabia [27], India [31], Chile [36], and Pakistan [28]. As a common complication affecting patients with T2DM, 3 studies were performed on patients with diabetes with renal abnormalities, including nephropathy or microalbuminuria [31,34,37], 1 study with diabetic foot ulcer [32], and 1 study with overweight condition [33]. The sex population consisted of males (*n* = 178), females (*n* = 43), and both sexes (*n* = 846). Our study population was all patients with T2DM aged between 46 to 66 and a total mean of 55. All studies were RCTs, of which 2 were single-blinded [27,30], 12 were double-blinded, and 1 was not clear. Three studies had cross-over designs [34,35,37], and 12 others were parallel. All 4 components of the lipid profile (LDL, HDL, TC, and TG) were assessed and reported in all studies, except for one study which did not include HDL in its trial [28]. The intervention was in 2 general forms of zinc supplement; 3 studies used the gluconate form [19,26,33] and the remaining 11 were in Sulfate form. Three studies cosupplemented zinc with α-linolenic acid [29], oral hypoglycemic agents [31], or multimineral vitamin [30], and one with metformin [38]. In the control group, we did not apply any restriction to be placebo, thus studies with cosupplementation in the intervention had the same compound in the control group [[29], [30], [31]]. The duration of the intervention ranged from 6 to 54 wk. Only 3 studies were performed for <12 wk [25,27,33], and the remaining 11 studies were ≥12 wk. Regarding the dosage of supplementation, we converted all of the dosages to elemental zinc content of supplements; 7 studies supplemented 30–50 mg daily elemental zinc [26,29,31,32,34,36,37], 2 studies were >50 mg daily [25,37], and 4 studies [27,28,30,33,35,38] were below 30 mg/d supplementation. We classified the studies based on baseline serum zinc according to a reference cutoff [39] of deficient (<70 μg/dL) and sufficient (>70 μg/dL). Two studies were categorized as deficient [26,30], and 8 were sufficient [19,29,[32], [33], [34], [35], [36], [37]]. Five studies did not report baseline serum zinc [25,27,28,31,38]. Except for 4 studies [25,27,28,31], adjustment for baseline serum zinc was implemented. In 5 studies, some variables were matched including lipid profile components, blood sugar indices, medication, sex, and age [26,28,31,32,34].

**TABLE 1:** Characteristics of randomized trials included in the systematic review on the eﬀects of zinc supplementation on lipid profiles^1^ in patients with type 2 diabetes mellitus

Author, y	Country	Health status	Sex	Age, y (mean ± SD)		Number		Study Design^2^	Intervention type		Duration (wk)	Dosage (elemental zinc mg/d)	Outcomes	Baseline serum zinc concentration (μg/dL)^3^	Change								Adjustment or matching
				Intervention	Control	Intervention	Control		Intervention	Control					Intervention Mean ± SD and number				Control Mean ± SD and number				
				Or Total											TG	LDL	HDL	TC	TG	LDL	HDL	TC	
Partida-Hernández, 2006 [35]	Mexico	T2DM	Male	51.70 ± 7.13		27	27	Ra/Db/Cr	sulfate	placebo	12	23	TG, TC, LDL, HDL	88	-29.67 ± 41.3	3.08 ± 72.18	18.45 ± 5.83	-18.77 ± 20.85	1.41 ± 26.6	6.42 ± 12.92	-7.48 ± 5.9	2.04 ± 18.73	No
Afkhami-Ardekani, 2008 [25]	Iran	T2DM	Both	52.67 ± 8.60		20	20	Ra/Db/Pa	sulfate	placebo	6	152	TG, TC, LDL, HDL	NR	-89.55 ± 70.8	-29.3 ± 27.64	7.3 ± 13.45	-34.65 ± 23.02	-8.85 ± 44	-3.25 ± 13.17	0.2 ± 8.62	96.95 ± 17.39	No
Parham, phase1, 2008 [34]	Iran	T2DM with microalbuminuria	Both	52 ± 9.3	54.5 ± 9.2	21	18	Ra/Db/Cr	sulfate	placebo	12	30	TG, TC, LDL, HDL	76	-10 ± 40	4 ± 79.79	-4 ± 5.37	-8 ± 16.98	2 ± 35	-7 ± 13.65	-4 ± 6.13	-11 ± 1367	all except TG
Parham, phase2, 2008 [34]	Iran	T2DM with microalbuminuria	Both	54.5 ± 9.2	52 ± 9.3	18	21	Ra/Db/Cr	sulfate	placebo	12	30	TG, TC, LDL, HDL	73	-23 ± 33.8	-3 ± 84.36	-2 ± 2.68	-10 ± 15.01	-32 ±31.4	-6 ± 11.99	-2 ± 3.13	-15 ± 14.12	all except TG
Seet, 2011 [19]	Singapore	T2DM	Male	57 ± 9	55 ± 8	20	20	Ra/Db/Pa	gluconate	placebo	12	120	TG, TC, LDL, HDL	88	7.49 ± 21.3	9.56 ± 91.09	-2.8 ± 4.71	-1.16 ± 15.61	0 ± 35	17.59 ± 26.77	0.4 ± 3.66	5.39 ± 24.87	Age
Gunasekara, 2011 [30]	Sri Lanka	T2DM	Both	54.1 ± 6	51.2 ± 6	29	31	Ra/Sb/Pa	sulfate +multivitamin/mineral	multivitamin/mineral	16	22	TG, TC, LDL, HDL	61	-1.76 ± 23.62	-2.3 ± 14.1	4 ± 5.90	-29.99 ± 16.60	-3.53 ± 72.18	-0.38 ± 12.83	1.6 ± 4.31	-5.39 ± 17.18	No
Ashmony, 2012 [27]	Saudi Arabia	T2DM	Both	48.46 ± 4.61	48.20 ± 4.09	26	30	Ra/Sb/Pa	sulfate	placebo cornstarch	8	10	TG, TC, LDL, HDL	NR	-21.85 ± 14.3	-33.77 ± 86.6	10.53 ± 3.22	-35.69 ± 7.14	14.50 ± 22.6	5.35 ± 6.67	-2.27 ± 6.39	16.73 ± 15.95	No
Foster, a, 2013 [29]	Australia	T2DM post-menopausal	Female	65.9 ± 10.8	64.6 ± 5.8	12	10	Ra/Db/Pa	sulfate	placebo	12	40	TG, TC, LDL, HDL	88	17.65 ± 7.9	0 ± 69.65	-4 ± 1.79	0 ± 5.16	8.83 ± 7.9	3.83 ± 5.13	-4 ± 1.79	3.83 ± 5.16	No
Foster, b, 2013 [29]	Australia	T2DM post-menopausal	Female	63.1 ± 5.1	66.2 ± 8.4	11	10	Ra/Db/Pa	sulfate + ALA	ALA	12	40	TG, TC, LDL, HDL	83	0 ± 7.89	3.82 ± 5.13	0 ± 1.79	7.69 ± 5.16	8.83 ±7.89	11.47 ± 5.13	0 ± 1.79	11.54 ± 5.16	No
Khan, 2013 [31]	India	T2DM with microalbuminuria	Both	56.3 ± 6.6	56.0 ± 8.6	23	21	Ra/NR/Pa	sulfate + OHA	OHA	12	50	TG, TC, LDL, HDL	NR	-43.87 ± 21.3	-12 ± 59.98	10.79 ± 2.98	-11.78 ± 8.33	21.2 ± 38	2.37 ± 11.37	0.12 ± 3.91	6.81 ± 11.94	FBS, PPbs, HbA1c, lipid, hs-CRP, age, duration, intake
Heravi, 2017 [32]	Iran	T2DM diabetic foot ulcer	Both	58.3 ± 8.6	60.0 ± 10.1	30	30	Ra/Db/Pa	sulfate	placebo	12	50	TG, TC, LDL, HDL	76,400	-7.9 ± 35.1	-10.8 ± 46.05	4.1 ± 3.54	-8.3 ± 20.22	-5.1 ± 21.2	-3 ± 11.39	1.2 ± 2.93	-3 ± 21.47	sex, medication, age
Pérez, 2018 [36]	Chile	T2DM	Both	55 ± 4.8	56 ± 8.1	13	15	Ra/Db/Pa	sulfate	placebo	54	30	TG, TC, LDL, HDL	8900	-2.4 ± 17.10	9.2 ± 79.44	-9.4 ± 7.6	2.2 ± 15.22	15.1 ± 19	13.8 ± 12.9	-0.4 ± 4.68	15.2 ± 13.71	All
Asghari, 2019 [26]	Iran	T2DM	Both	46.2 ± 5.3	45.5 ± 5.4	30	30	Ra/Db/Pa	gluconate	placebo	12	30	TG, TC, LDL, HDL	71	-17.10 ± 44.2	-0.9 ± 77.02	5.3 ± 9.18	-2.83 ± 21.76	-18.3 ± 37.3	-3 ± 11.39	-1.5 ± 3.36	-8.2 ± 20.24	sex, age, medications, HTN, diabetes duration
Nazem, 2019 [33]	Iran	T2DM overweight	Both	53.28 ± 7.35	54.34 ± 7.18	35	35	Ra/Db/Pa	gluconate	Placebo	8	7	TG, TC, LDL, HDL	95	-6.38 ± 21.3	-5.65 ± 56.85	4.6 ± 3.55	-11.85 ± 14.41	17.35 ± 23.8	5.12 ± 8.04	1.2 ± 4.01	-3.11 ± 13.32	All
Farooq, 2020 [28]	Pakistan	T2DM	Both	51.21+10.115	51.21+10.115	175	175	Ra/Db/Pa	sulfate	Placebo	12	7	TG, TC, LDL	NR	-4.16 ± 19.84	-3.85 ± 14.96	-	-2.91 ± 16.16	-0.04 ± 19.15	0 ± 10.98	-	0 ± 10.62	age, sex
Sharifi, phase1, 2022 [38]	Iran	T2DM with nephropathy	Male	NR	NR	21	21	Ra/Db/Pa	sulfate	Placebo	12	30	TG, TC, LDL, HDL	102.5	0.10 ± 31.9	-6.6 ± 70.8	1 ± 1.29	-5 ± 15.14	-15 ± 52.8	-1.8 ± 13.35	0.2 ± 1.12	-4.6 ± 14.27	All
Sharifi, phase2, 2022 [38]	Iran	T2DM with nephropathy	Male	NR	NR	21	21	Ra/Db/Pa	sulfate	Placebo	12	30	TG, TC, LDL, HDL	99	-3.9 ± 27.4	-3.7 ± 71.5	1.2 ± 1.88	-3.3 ± 14.18	-2.60 ± 23.7	-3.7 ± 12.46	1 ± 1.07	-3.3 ± 13.74	All
Younis, 2021 [39]	Iraq	T2DM	Both	47.5 ± 8.95	30.37 ± 3.46	35	32	NR/NR/NR	gluconate + metformin	metformin	8	7	TG, TC, LDL, HDL	68,600	-13.7 ± 26.2	-22.6 ± 13.41	2.66 ± 2.27	-20.4 ± 14.92	8.6 ± 25.9	-3.4 ± 20.95	0.17 ± 2.51	-1.9 ± 22.38	No

FBS,; HbA1c, glycated hemoglobin; hsCRP, high-sensitivity C-reactive protein; HTN, hypertension; PPbs,; T2DM, type 2 mellitus.

1 Total cholesterol (TC), triglyceride (TG), LDL, HDL.

2 Randomized (Ra), double-blinded (Db), parallel (Pa), cross-over (Cr), single-blinded (Sb).

3 Not reported (NR).

#### Risk of bias assessment

The results of quality assessment according to Cochrane Collaboration’s risk of bias tool are presented in Table 2. The quality assessment discovered that 7 studies were of good quality [19,26,29,32,[35], [36], [37]], 3 were fair [25,33,34], and 5 were poor [27,28,30,31,38]. Bias was evaluated based on adequate sequence generation, allocation concealment, participant and personnel blinding, outcome assessment blinding, incomplete data, and selective reporting as well as other possible sources of bias.

**Table 2:** Cochrane risk of bias assessment

Study, year	Random sequence generation	Allocation concealment	Blinding of participants and personnel	Blinding of outcome assessment	Incomplete outcome data	Selective outcome reporting	Other sources of bias	Overall quality
Partida-Hernández, 2006 [35]	L	L	L	L	L	L	L	good
Afkhami-Ardekani, 2008 [25]	L	L	L	L	L	UC	L	fair
Parham, 2008 phase1 [34]	L	L	L	L	L	UC	L	fair
Seet, 2011 [19]	L	L	L	L	L	L	L	good
Gunasekara, 2011 [30]	L	L	L	H	H	L	L	poor
Ashmony, 2012 [27]	L	L	L	L	H	UC	L	poor
Foster, 2013 [29]	L	L	L	L	L	L	L	good
Khan, 2013 [31]	L	L	L	H	UC	UC	L	poor
Heravi, 2017 [32]	L	L	L	L	L	L	L	good
Pérez, 2018 [36]	L	L	L	L	L	L	L	good
Asghari, 2019 [26]	L	L	L	L	L	L	L	good
Nazem, 2019 [33]	L	L	L	L	UC	UC	L	fair
Farooq, 2020 [28]	L	L	H	L	L	L	L	poor
Sharifi, 2022 [38]	L	L	L	L	L	L	L	good
Younis, 2021 [39]	H	H	L	L	UC	UC	UC	poor

H, high risk of bias; L, low risk of bias; UC, unclear risk of bias.

### Findings from meta-analysis

#### Effects of zinc on TC

**FIGURE 2:**
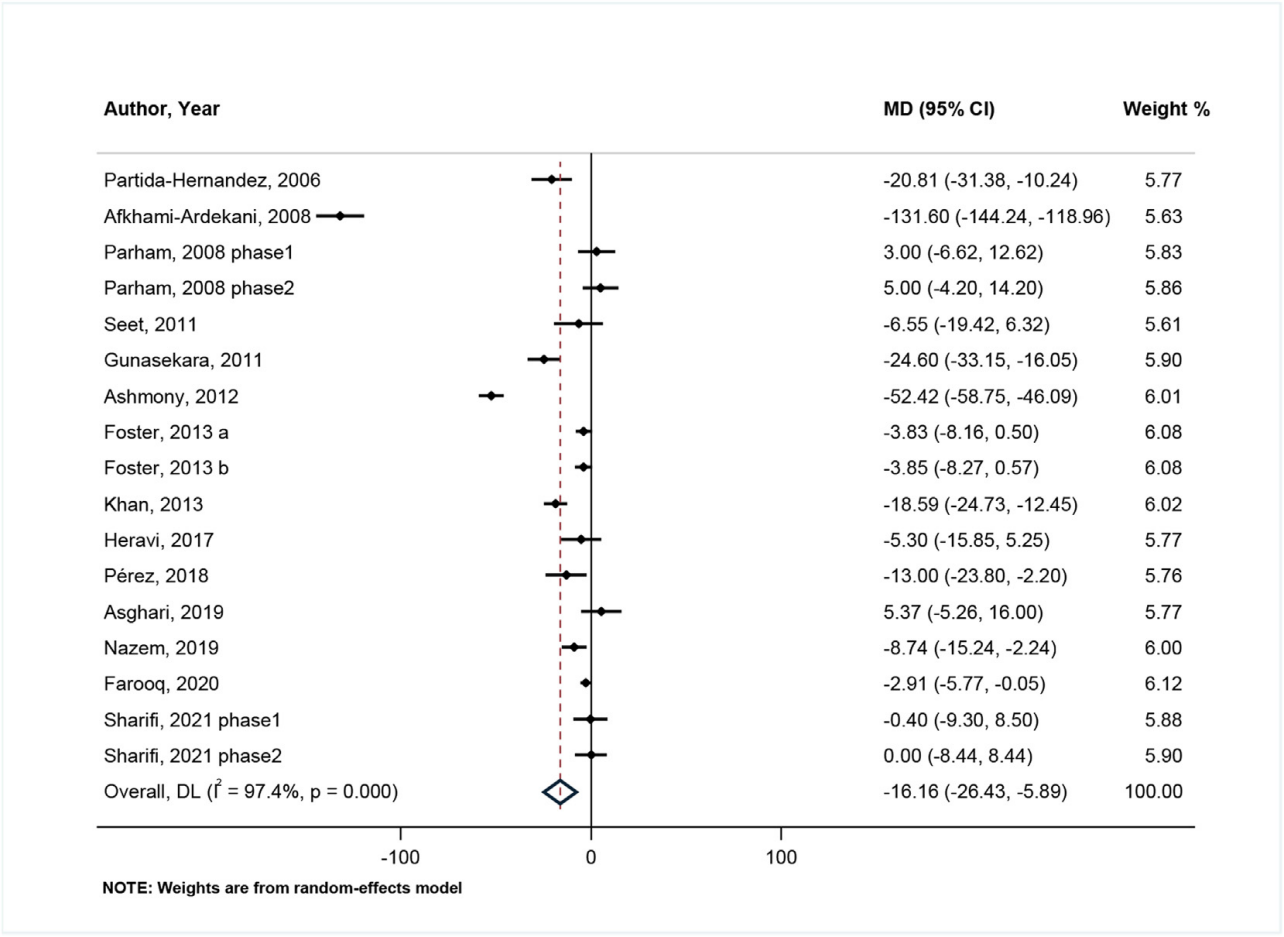
Forest plot for comparison of zinc supplementation with placebo/no zinc from baseline to postintervention for serum total cholesterol (μg/dL) in patients with type 2 diabetes mellitus. MD (95% CI) = Mean Differences comparing before and after changes between intervention and placebo groups with 95% confidence interval. DL, DerSimonian & Laird method for random-effect meta-analysis.

After combining the findings of 14 studies with 17 effect sizes, a significant reduction in serum concentrations of TC was observed after zinc supplementation (weighted mean difference [WMD]: −16.16; 95% CI: −26.43, −5.89; *P* = 0.002) (Figure 2). There was significant between-study heterogeneity (I^2^ = 97.4%, *P* < 0.001). Based on the findings from subgroup analysis, study design, sex, country, age, health condition, duration, matching for variables, adjustment for baseline serum zinc, baseline serum zinc, and study quality were assumed to be potential sources of heterogeneity (Supplementary Table 1). In all subgroups, the reduction effect of zinc on TC remained significant.

According to the results of the sensitivity analysis, the main result remained significant and stable after the exclusion of every single trial from the analysis, ranging from −17.48 mg/dL (95% CI: −28.18, −6.78) to −9.34 mg/dL (95% CI: −16.38, −2.31). No significant publication bias was observed in both Begg (*P* = 0.174) and Egger tests (95% CI: −11.58, 2.70; *P* = 0.205) or with the assessment of the funnel plot (Supplementary Figure 1C).

#### Effects of zinc on HDL

**FIGURE 3:**
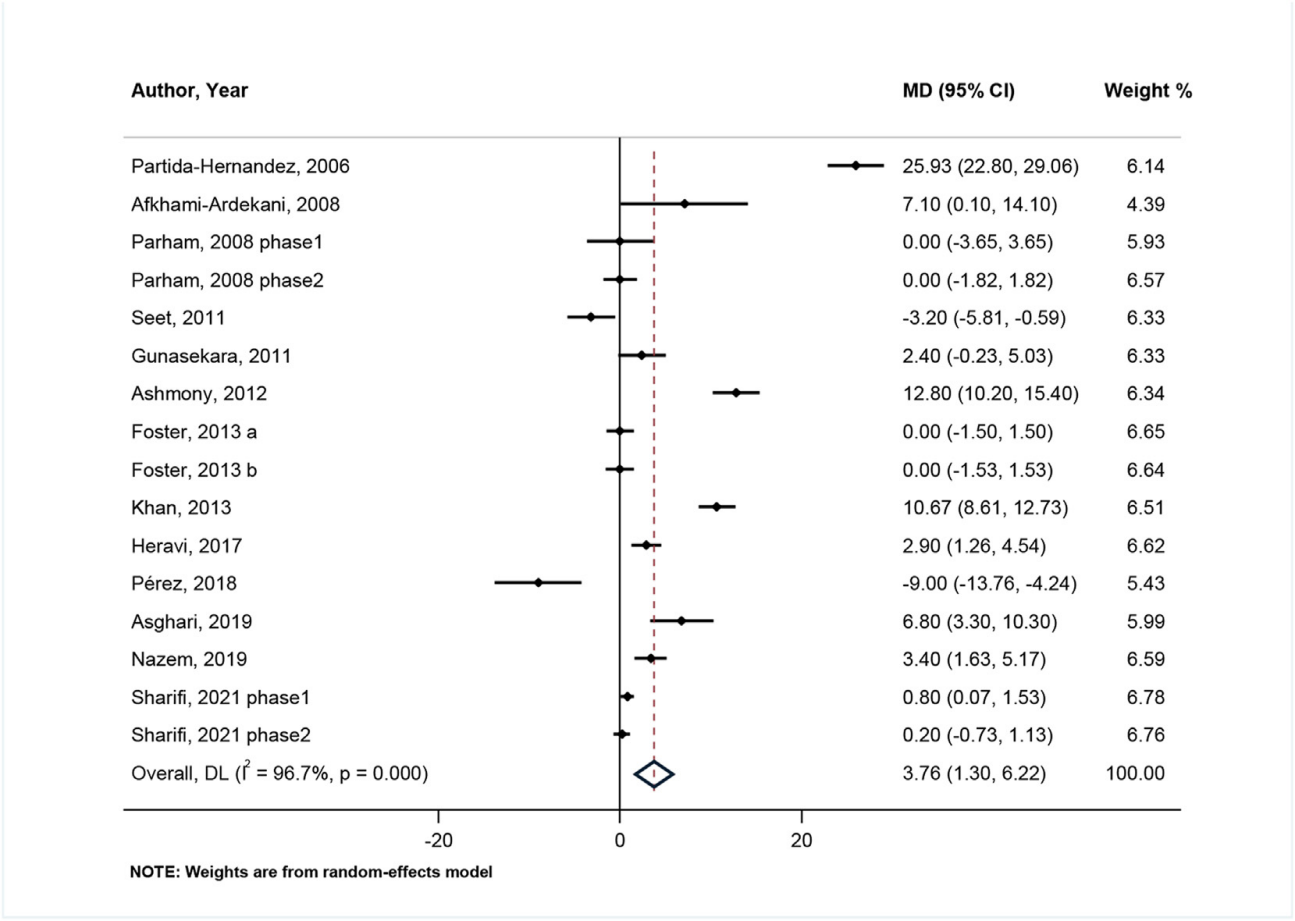
Forest plot for comparison of zinc supplementation with placebo/no zinc from baseline to postintervention for serum HDL (μg/dL) in patients with type 2 diabetes mellitus.MD (95% CI) = Mean Differences comparing before and after changes between intervention and placebo groups with 95% confidence interval. DL, DerSimonian & Laird method for random-effect meta-analysis.

There were 13 clinical trials with 16 effect sizes that examined the effects of zinc supplementation on serum HDL. A significant elevation in serum concentrations of HDL was seen after zinc supplementation (WMD: 3.76; 95% CI: 1.30, 6.22; *P* = 0.003) (Figure 3). Significant heterogeneity was detected between studies (I^2^ = 96.7%, *P* < 0.001). The source of heterogeneity was defined by subgroup analysis. Study design, sex, country, age, health condition, intervention and control type, duration, matching for variables, adjustment for baseline serum zinc, baseline serum zinc status, and study quality were assumed to be potential sources. Except for female sex and age >60, for all subgroups the increment effect of zinc on HDL remained significant (female: WMD: 0.00; 95% CI: −1.07, 1.07; age >60 y: WMD: 0.86; 95% CI: −0.03, 1.76) (Supplementary Table 2).

Regarding the findings of the sensitivity analysis, none of the studies had a significant single effect and did not affect the final result and ranged from 2.31 mg/dL (95% CI: 0.48, 4.14) to 4.48 mg/dL (95% CI: 2, 6.97). According to none of Begg (*P* = 0.192) and Egger tests (95% CI: −1.58, 9.46; *P* = 0.148), no significant publication bias was observed, as demonstrated in the funnel plot (Supplementary Figure 1A).

#### Effects of zinc on LDL

**FIGURE 4:**
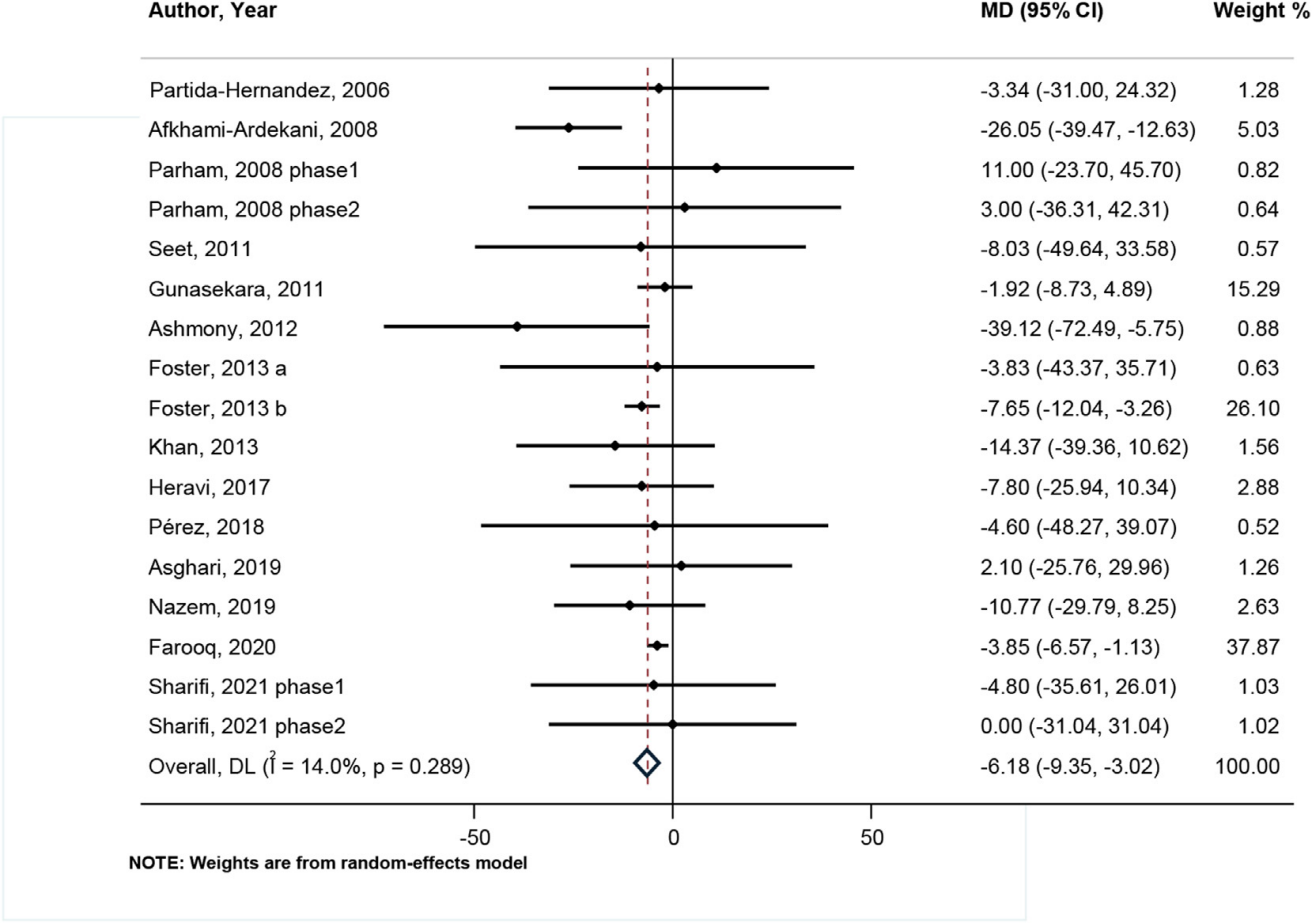
Forest plot for comparison zinc supplementation with placebo/no zinc from baseline to postintervention for serum low-density lipoprotein (μg/dL) in patients with type 2 diabetes mellitus. MD (95% CI) = Mean Differences comparing before and after changes between intervention and placebo groups with 95% confidence interval. DL, DerSimonian & Laird method for random-effect meta-analysis.

After analyzing the results of 14 studies with 17 effect sizes, a significant reduction in serum concentrations of LDL was observed following zinc supplementation (WMD: −6.18; 95% CI: −9.35, −3.02; *P* < 0.001) (Figure 4). In contrast with other lipid profiles, no significant heterogeneity was observed between studies (I^2^ = 14.0%, *P* = 0.289), and in almost all subgroups, the reductive effect of zinc supplementation on LDL remained significant (Supplementary Table 3).

We observed that through performing the sensitivity analysis, none of the studies had a more noticeable distinctive effect than other studies on the final result, and it ranged from −7.56 mg/dL (95% CI: 11.48, −3.65) to −5.16 mg/dL (95% CI: −7.24, −3.07). We did not detect any significant publication bias in either Begg (*P* = 0.902) or Egger tests (95% CI: −1.04, 0.40; *P* = 0.357), the same as the funnel plot publication bias assessment tool (Supplementary Figure 1B).

#### Effects of zinc on TG

**FIGURE 5:**
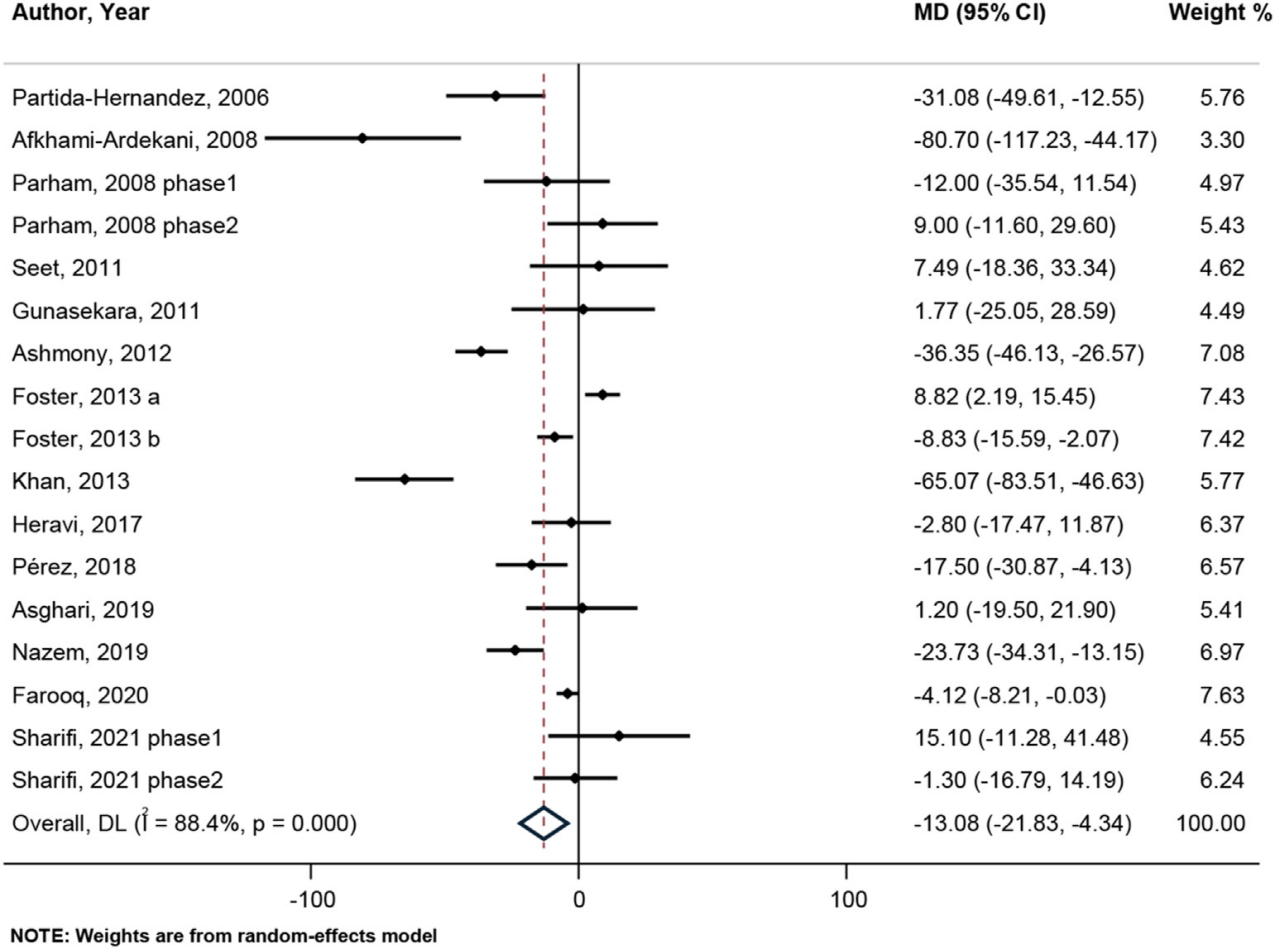
Forest plot for comparison zinc supplementation compared with placebo/no zinc from baseline to postintervention for serum triglycerides (μg/dL) in patients with type 2 diabetes mellitus. MD (95% CI) = Mean Differences comparing before and after changes between intervention and placebo groups with 95% confidence interval. DL, DerSimonian & Laird method for random-effect meta-analysis.

After analyzing the results of 14 studies with 17 effect sizes, a significant reduction in serum concentrations of TG was observed following zinc supplementation (WMD: −13.08; 95% CI: −21.83, −4.34; *P* = 0.003) (Figure 5). Because a significant heterogeneity was observed among studies (I^2^ = 88.4%, *P* < 0.001), we recourse to findings in subgroup analysis and found that study design, sex, country, age, intervention and control type, duration, adjustment for baseline serum zinc, baseline serum zinc status, and study quality are the possible sources of heterogeneity. Almost in all subgroups, the reductive effect of zinc supplementation remained significant. (Supplementary Table 4).

It is acknowledged that after removing each study individually in sensitivity analysis, none of the studies had a more prominent unique effect than the other studies, and the weighted mean differences ranged from −14.83 mg/dL (95% CI: −23.76, −5.90) to −9.81 mg/dL (95% CI: −17.76, −1.87). Based on none of Begg (*P* = 0.967) and Egger test (95% CI: −4.14, 1.10; *P* = 0.237), there was no significant bias in the publication, as evaluated by the funnel plot tool (Supplementary Figure 1D).

### Findings from dose–response analysis

#### TC

**FIGURE 6:**
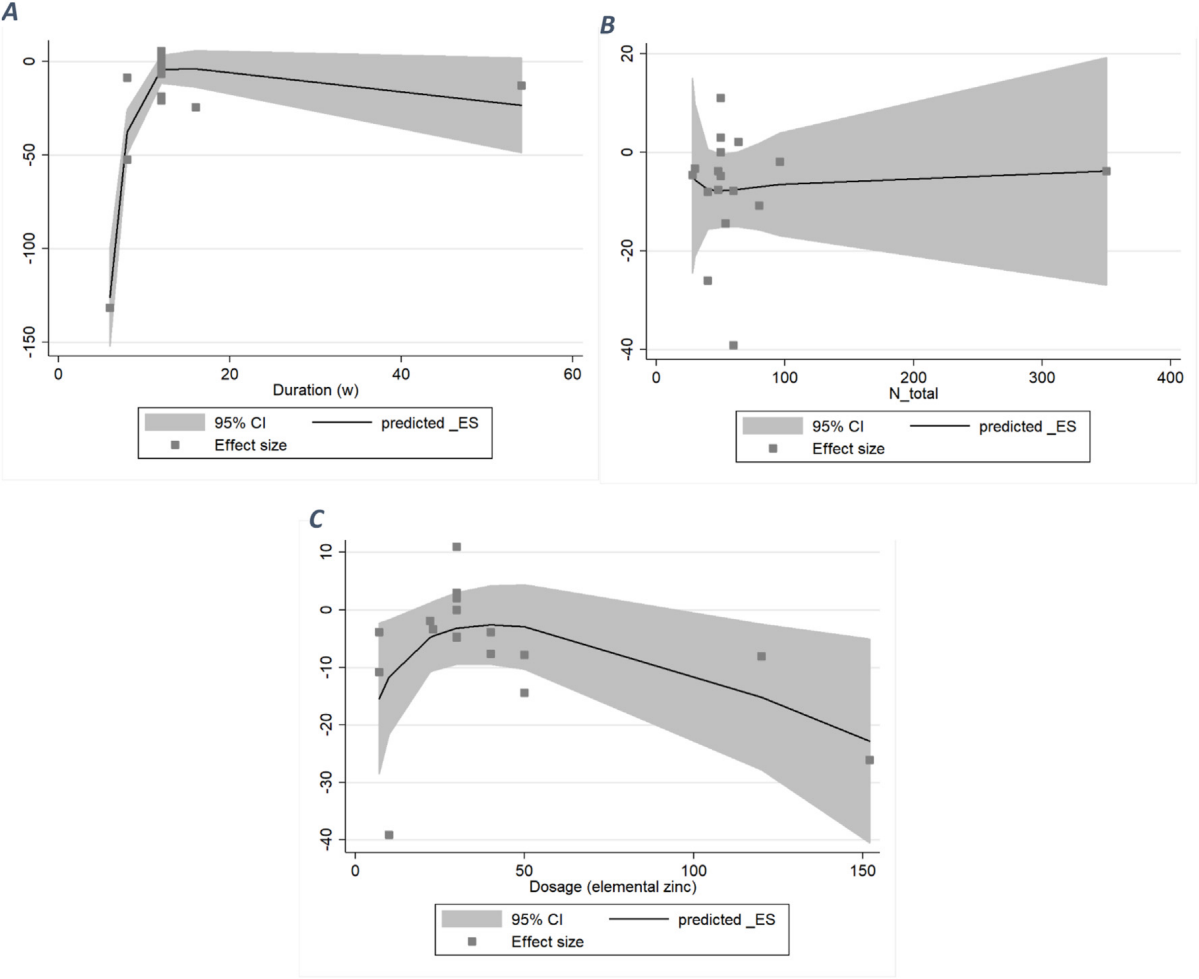
A dose–response meta-analysis of changes in total cholesterol (μg/dL) according to zinc supplementation in the treatment and control groups at the end of the trials (all studies) and by the duration of intervention and total population. The average curve (solid line) with 95% confidence limits (dashed lines) was estimated with a 1-stage random-effects restricted cubic spline model.

Following a nonlinear dose–response analysis, a significant inverse association was observed between <12 wk zinc supplementation and serum TC (WMD: −5, *P*_nonlinearity_ < 0.001). Also, an inverse relation was seen between >120 mg/d elemental zinc supplementation and serum TC (WMD: −5, *P*_nonlinearity_ < 0.001). The association between the number of total participants was neither nonlinear nor significant (*P*_nonlinearity_ < 0.758) (Figure 6).

#### HDL

**FIGURE 7:**
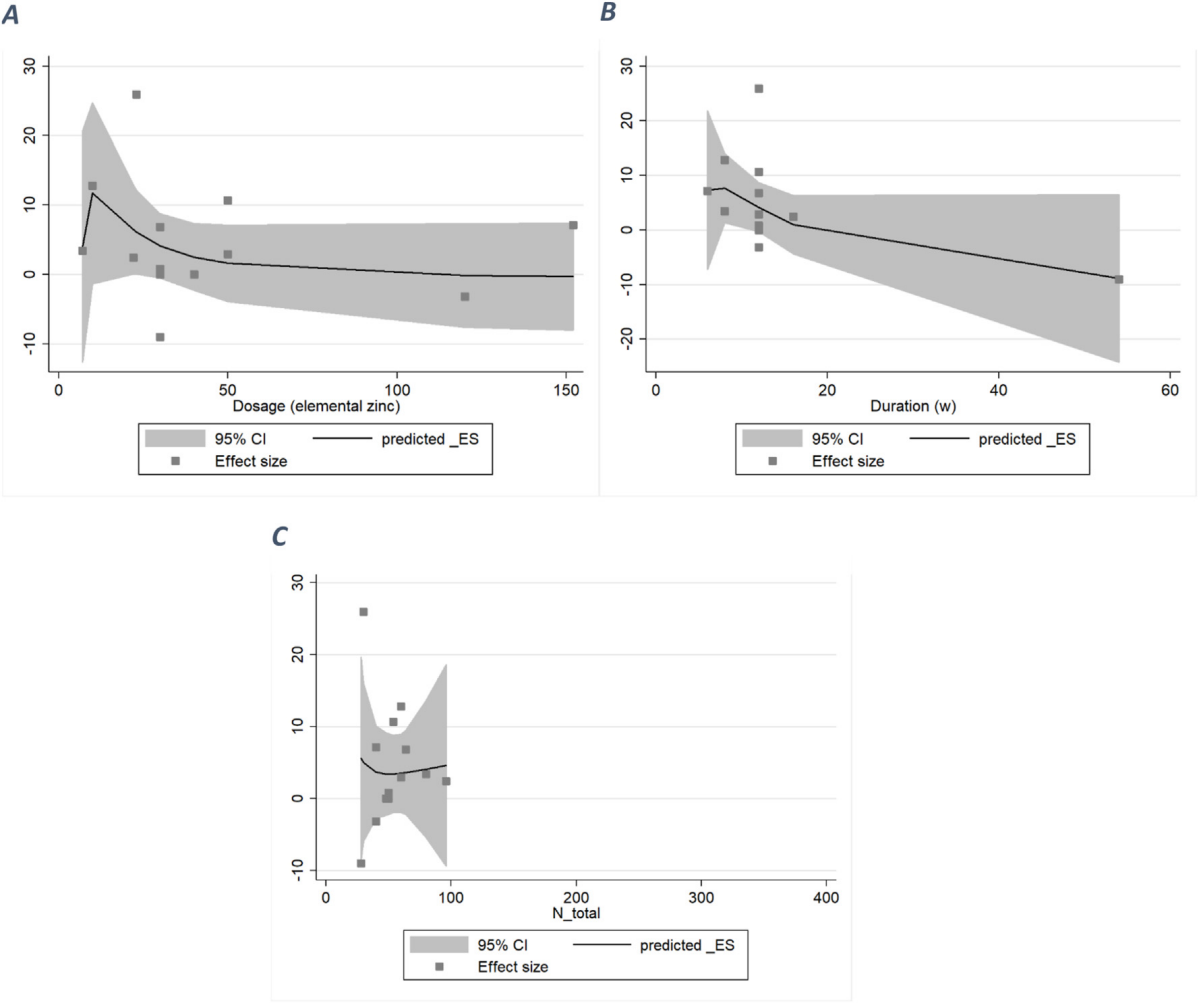
A dose–response meta-analysis of changes in HDL (μg/dL) according to zinc supplementation in the treatment and control groups at the end of the trials (all studies) and by duration of intervention and total population. The average curve (solid line) with 95% confidence limits (dashed lines) was estimated with a 1-stage random-effects restricted cubic spline model.

After performing a nonlinear dose–response analysis, no significant association was observed between both the duration and dosage of zinc supplementation and serum HDL (duration: *P*_nonlinearity_ = 0.406; dosage: *P*_nonlinearity_ = 0.188). Similarly, there was no significant association between dosage or duration and HDL serum concentration (Figure 7).

#### LDL

**FIGURE 8:**
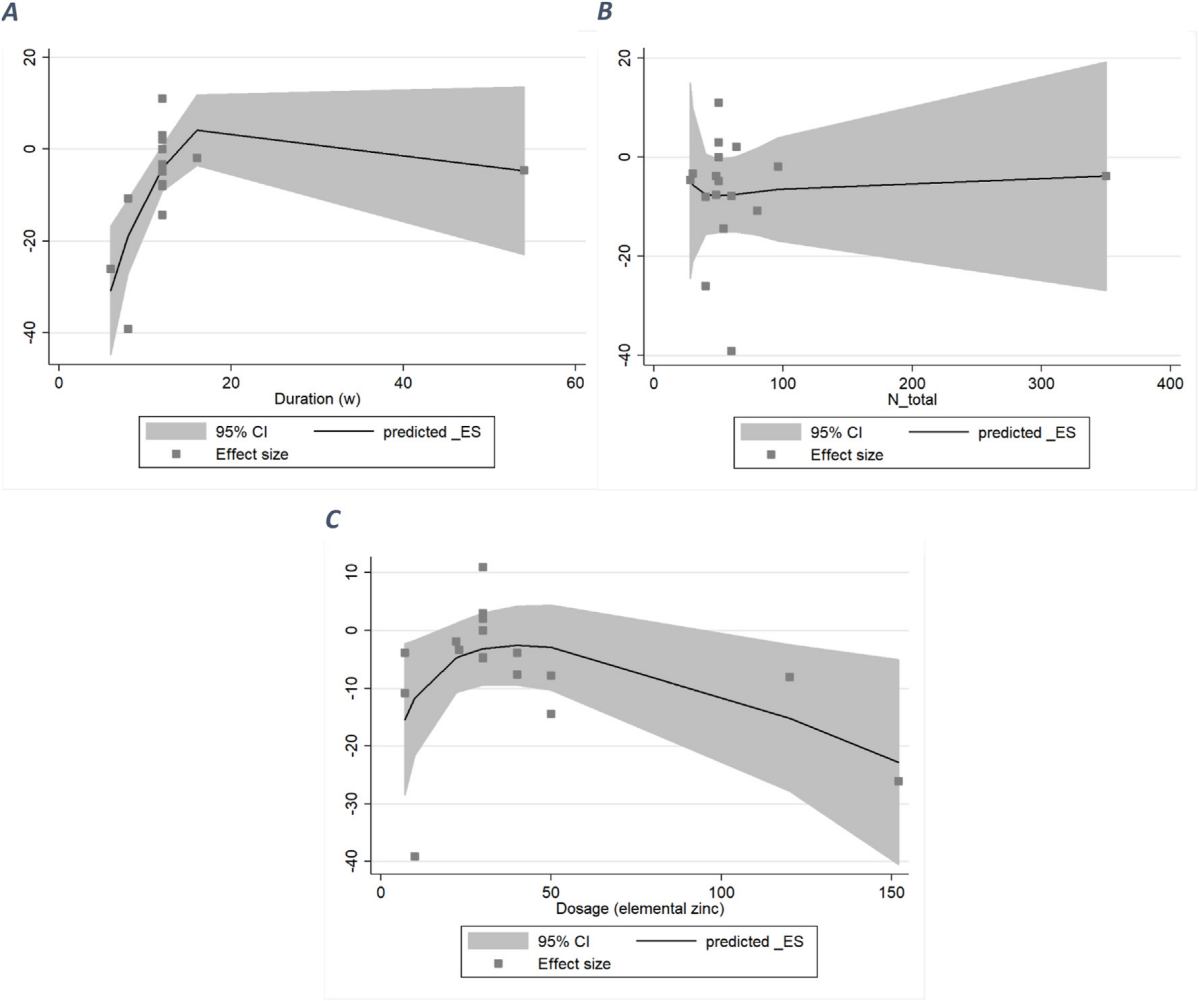
A dose–response meta-analysis of changes in low-density lipoprotein (μg/dL) according to zinc supplementation in the treatment and control groups at the end of the trials (all studies) and by duration of intervention and total population. The average curve (solid line) with 95% confidence limits (dashed lines) was estimated with a 1-stage random-effects restricted cubic spline model.

After performing a nonlinear dose–response analysis, a significant inverse association was observed between <12 wk of zinc supplementation and serum LDL (WMD: −5, *P*_nonlinearity_ = 0.07). Also, an inverse relation was seen between >100 mg/d elemental zinc supplementation and serum LDL (WMD: −10, *P*_nonlinearity_ = 0.006). The association between the number of total participants was neither nonlinear nor significant (*P*_nonlinearity_ = 0.925) (Figure 8).

#### TG

**FIGURE 9:**
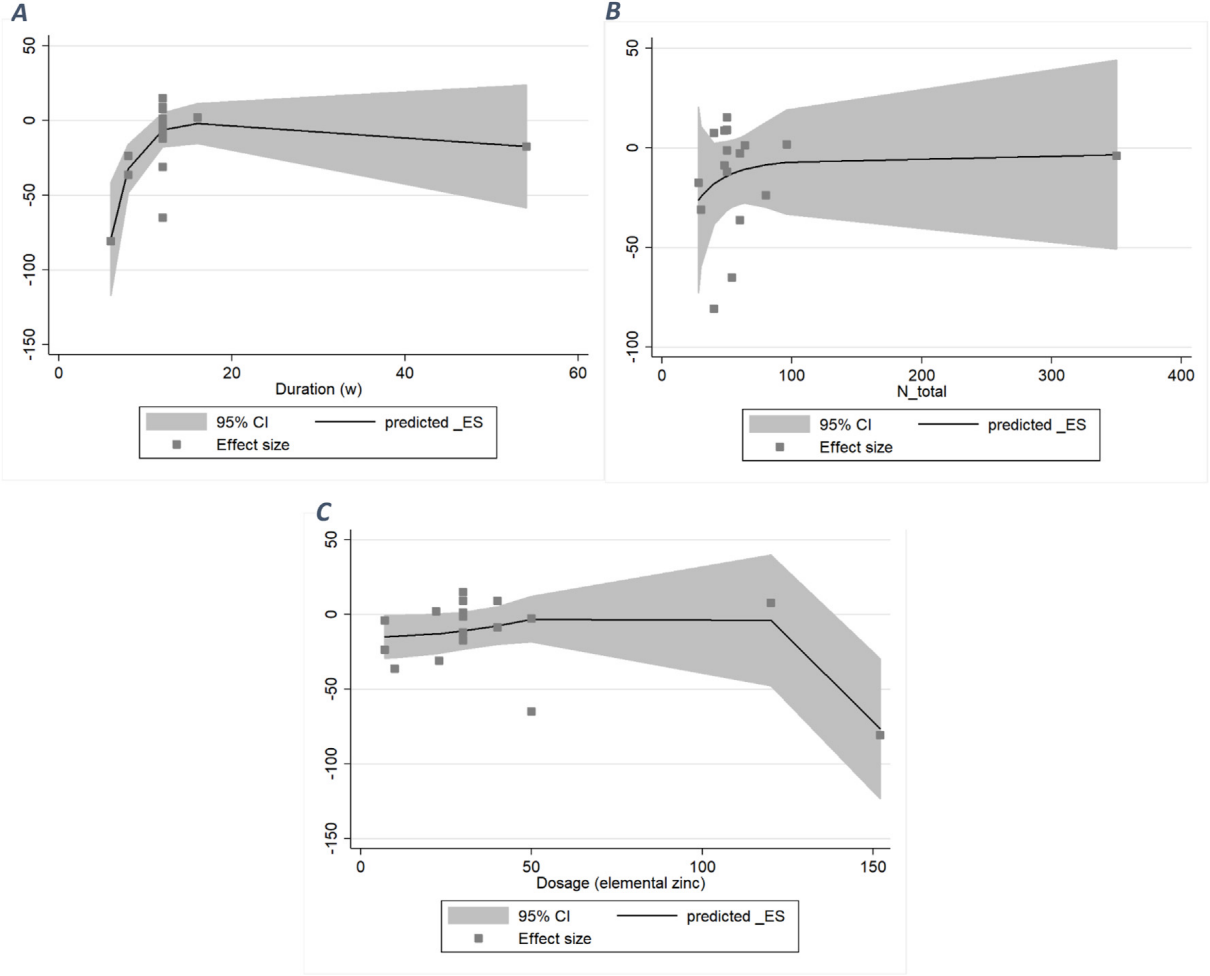
A dose–response meta-analysis of changes in triglycerides (μg/dL) according to zinc supplementation in the treatment and control groups at the end of the trials (all studies) and by duration of intervention and total population. The average curve (solid line) with 95% confidence limits (dashed lines) was estimated with a 1-stage random-effects restricted cubic spline model.

After performing a nonlinear dose–response analysis, both duration and dose had a nonlinear relation with TG concentration (dose: *P*_nonlinearity_ = 0.031; duration: *P*_nonlinearity_ = 0.006). A significant inverse association was observed between <12 wk of zinc supplementation and serum LDL (WMD: −16.5). Also, an inverse relation was seen between >140 mg/d elemental zinc supplementation and serum TC (WMD: −50). The association between the number of total participants was neither nonlinear nor significant (*P*_nonlinearity_ = 0.724) (Figure 9).

## Discussion

This meta-analysis was conducted to investigate the effects of zinc on the lipid profile indices LDL, HDL, TG, and TC. A total of 14 RCTs on the impacts of different sorts of elemental zinc supplementation on lipid profile in patients with T2DM were included in our meta-analysis. We showed that zinc supplementation has significantly improved all 4 components of the lipid profile in the T2DM population. Above all, we implemented dose–response analysis for all 4 lipid profile components based on the dose, duration, and population of the interventions. We found that a 12-wk period of intervention was the highest duration to supplement zinc for TC, TG, and LDL. The lowest necessary dose of a zinc supplement to create improvement effects in serum lipid profile concentrations is 120, 100, and 140 mg daily, respectively for LDL, TC, and TG. In summary, the optimum dose and duration for zinc supplementation to improve serum lipid profile concentrations are 140 mg daily and ≤12 wk, respectively. We acknowledge that the upper tolerable limit for zinc intake is established to prevent adverse effects, particularly mineral absorption [40]. Thus, to avoid interaction with the absorption of other minerals and nutrients, taking zinc supplements at intervals from other supplements and main meals can be an effective measure in preventing the malabsorption of other minerals [41,42]. However, none of the studies included in our analysis reported adverse events or complications of toxicity because of zinc supplementation. Studies have shown that pharmacologic doses of zinc, which range between 100 and 300 mg daily, are standard clinical recommendation doses for short-term practices [43]. Compared with the tolerable upper limit and lowest-observed-adverse-effect concentration of zinc ranging from 40 to 60 mg daily [44,45], the pharmacologic dose of zinc supplement will not cause people to over intake zinc and cause toxic effects, especially in the short term [16]. The suggested dose is obtained from the dose–response and based on their significant effectiveness. This dose of zinc supplement may be higher than the daily intake, which can indicate that to create significant effects on the level of the desired factors in patients with diabetes, a higher dose than healthy people is needed. However, this dose is also within the common pharmaceutical dose range.

After performing subgroup analysis to find sources of heterogeneity, the remaining components had a significant heterogeneity except for LDL. Most subgroups did not change the significance of the findings; however, in the sex subgroup for TG, we found that studies with unique sexes and studies performed on populations aged >60 y were out of significance. As another example, baseline serum zinc status was a heterogeneous source for TG and HDL.

Previous findings on the effects of zinc supplementation on lipid profile are conflicting. According to Seet et al. [19], zinc supplementation increased TG and LDL concentrations and decreased HDL. It should be clarified that the baseline serum zinc concentrations might affect these findings. In our study, based on the subgroup analysis, we showed that the baseline zinc status has a significant effect on the result of the supplementation, such that the improvement of the lipid profile was greater in the target population at baseline. According to the study of Al-Maroof et al. [47], the zinc concentration in diabetic patients is greatly affected by their disease, and the results of supplementation were also significantly different from the healthy control group. Therefore, zinc supplementation is more effective in patients with diabetes if they have lower baseline serum zinc concentrations.

Diverse types of zinc supplements had no general significantly different effects than the overall effect. As shown in the subgroup analysis (Supplementary Tables 1–4), the between-group analysis for intervention type was not significant either for TC (*P*_between-subgroup_ = 0.123) or LDL (*P*_between-subgroup_ = 0.827), in contrast with HDL (*P*_between-subgroup_ = 0.005) and TG (*P*_between-subgroup_ = 0.009), whereas the reduction in effect size for the gluconate type was more noticeable.

Contradictory shreds of evidence in HDL concentrations are more common. Four trials showed a decrease in HDL concentrations [19,29,34,36]. This may be because most of these studies were performed on specific sexes and not on both, which appeared to be one of our sources of heterogeneity in subgroup analysis. Similarly, it was found in the case of TG in the subgroup analysis, the reducing effect of zinc had a nonsignificant opposite result for the female subgroup (WMD: 0.16, 95% CI: −4.57, 4.89). An important reason for the conflicting results of primary studies with our meta-analysis is the small number of participants, so when we put the total number of studies together in our analysis, the final and significant effect size is more reliable.

An earlier meta-analysis by Asbaghi et al. [48] published in 2020 showed a distinguishable improvement in TC and TG but not in LDL and HDL. Another meta-analysis by Pompano et al. [49] published in 2021 showed benefits in both low and high dose-duration supplementations. However, there are limitations involved in the mentioned meta-analysis. Pompano’s study was not limited to patients with T2DM, and the health condition of included studies varied from healthy, pediatric, and pregnant to polycystic ovary syndrome and T2DM, which made it hard to conclude any recommendations on zinc supplementation for specific health conditions or population characteristics. Also, Asbaghi et al. [48] did not include 3 RCTs [26,30,31]. One study was excluded due to the mixed nature of the control group [31], and the other 2 studies probably were not found in their systematic search [26,30]. Moreover, since the latest meta-analysis, 3 clinical trials were performed and published [28,37,38], which allows us to update the previous reviews. Contrary to the latest meta-analysis by Asbagi et al. [48], which failed to find a significant effect of zinc supplementation on either LDL or HDL in patients with T2DM, we found zinc supplementation effective on both LDL and HDL similar to TC and TG. Similar to the previous meta-analysis, TG and TC met significant improvements, and in contrast with the abovementioned study, LDL and HDL also showed significant advances. This may be because the meta-analysis of Asbaghi et al. [48] did not include the aforementioned 6 studies. Totally, as the privilege of our research over their study, we included 5 more trials with 8 more effect sizes in our statistical analysis, which have allowed us to perform a more comprehensive review. Studies have also been conducted on other populations that were in accord with our findings, including healthy obese adults, patients poorly controlled with metformin, or hypertensive patients [[50], [51], [52]].

A possible mechanism to assume for this would be related to zinc's effects on glucose and lipid metabolism. Zinc inhibits hormone-sensitive lipase through a 3-kinase–Akt/PKB signaling cascade by complexes dependent on the phosphoinositide. Regarding these findings, it is hypothesized that zinc supplementation results in both improved glucose utilization and lipid metabolism [53]. Also, it has been investigated that zinc cooperates in lipoprotein lipase and lecithin cholesteryl ester transferase pathways; thus, it is proposed that *ZNF202* may be a probable gene vulnerable to developing dyslipidemia in human body [54]. Also, by participating in the construction of an adipokine called zinc-α2-glycoprotein, zinc increases the secretion of adiponectin and inhibits the secretion of leptin in human body. If the metabolism of zinc is disturbed, the regulatory role of zinc on these adipokines is defective. Therefore, the chance of dyslipidemia and metabolic syndrome diseases increases [55].

To our knowledge, being the first meta-analysis to examine the effects of zinc supplements on the lipid profile of patients with T2DM considering the multiple dose–response analysis is our main study strength. However, here are some limitations attributed to our study. Despite many efforts to obtain the full text of the articles, 1 article was not found to be used in the extraction and analysis [56]. Another weakness of our work was putting together different types of zinc supplements. However, in the data extraction stage, we converted the amount of zinc from different supplements to elemental zinc, and for this reason, we did not observe heterogeneity between different types of zinc supplements in the subgroup analysis. Considering the Recommended Dietary Allowance of zinc and its upper limit in the recommended dose of the dose–response analysis, in our study, there was not enough evidence in the included RCTs because none reported adverse events. Side effects due to the use of the supplement should be investigated in future studies to ensure the safety and efficacy of zinc supplementation.

In conclusion, zinc supplementation significantly improved lipid profile in patients with T2DM. The findings of our research can help practitioners in the process of treating and improving the complications caused by T2DM, however, our findings are mostly aimed at patients with low serum zinc concentrations and short-term zinc supplementation.

### Author contributions

The authors’ responsibilities were as follows – LA, MH-S, SE-K: designed the study; MH-S, MM: conducted the literature search and screening of published papers; MH-S, MM: performed the data extraction and quality assessment, independently; MH-S, SE-K: performed the statistical analysis; MH-S, MM: interpreted data and wrote the manuscript; LA: supervised the study; and all authors: read and approved the final manuscript.

### Conflict of interest

The authors report no conflicts of interest.

### Funding

This study was supported by Tehran University of Medical Sciences (grant number: 1401-3-212-63084)

### Data availability statement

Data sharing is not applicable.

## Appendix A Supplementary data

Mutimedia component 1 Mutimedia component 1
