# Supplementary material for: The Effect of Zinc Supplementation on Lipid Profiles in Patients with Type 2 Diabetes Mellitus: A Systematic Review and Dose–Response Meta-Analysis of Randomized Clinical Trials
Source: Adv Nutr. 2023 Aug 19;14(6):1374–88. doi: 10.1016/j.advnut.2023.08.006 (PMC10721485; doi:10.1016/j.advnut.2023.08.006)
Supplement: Mutimedia component 1 [file mmc1.docx]

**The effect of zinc supplementation on lipid profile in Type-2 diabetes mellitus patients: A systematic review and dose-response meta-analysis of randomized clinical trials**

First Author: Mohammad Heidari Seyedmahalleh

**Supplementary tables**

| Group | Comparisons, n | WMD (95% CI) | P-value^1^ | I^2^ (%) | P-heterogeneity^2^ |
| --- | --- | --- | --- | --- | --- |
| **Total** | **17** | **-16.284 (26.112, -6.455)** | **0.000** | **97.3** |  |
| **Study Design** |  |  |  |  | **0.000** |
| Randomized | 3 | -32.79 (-36.71, -28.87) | 0.000 | 96.7 |  |
| Randomized Blinded | 5 | -1.71 (-5.85, 2.43) | 0.003 | 74.6 |  |
| Parallel Randomized Blinded | 9 | -6.70 (-8.57, -4.84) | 0.000 | 98.0 |  |
| **Sex** |  |  |  |  | **0.000** |
| Male | 4 | -5.54 (-10.44, -0.64) | 0.012 | 72.7 |  |
| Female | 2 | -3.84 (-6.93, -0.74) | 0.995 | 0.0 |  |
| Both | 11 | -13.318 (-15.257 , -11.379) | 0.000 | 98.3 |  |
| **Country** |  |  |  |  | **0.000** |
| Developed | 2 | -3.84 (-6.93, -0.74) | 0.995 | 0.0 |  |
| Developing | 15 | -12.266 (-14.069 , -10.462) | 0.000 | 97.7 |  |
| **Age** |  |  |  |  | **0.000** |
| ≤60 years | 10 | -10.557 (-12.531 , -8.584) | 0.000 | 96.3 |  |
| >60 years | 3 | -3.95 (-6.92, -0.98) | 0.967 | 0.0 |  |
| Not Reported | 4 | -24.23 (-29.12, -19.34) | 0.000 | 99.1 |  |
| **Health Condition** |  |  |  |  | **0.004** |
| With micro albuminuria /nephropathy | 5 | -5.36 (-9, -1.73) | 0.000 | 85.9 |  |
| Without micro albuminuria /nephropathy | 12 | -11.201 (-12.926 , -9.477) | 0.000 | 98.1 |  |
| **Interventions Type** |  |  |  |  | **0.123** |
| Sulfate | 11 | -10.44 (-12.33, -8.56) | 0.000 | 98.3 |  |
| Gluconate | 3 | -5.16 (-10.25, -0.06) | 0.083 | 59.8 |  |
| Mixed | 3 | -11.234 (-14.541 , -7.927) | 0.000 | 92.2 |  |
| **Control Type** |  |  |  |  | **0.457** |
| Placebo | 14 | -9.81 (-11.57, -8.04) | 0.000 | 97.8 |  |
| Non-Placebo | 3 | -11.234 (-14.541 , -7.927) | 0.000 | 92.2 |  |
| **Duration** |  |  |  |  | **0.000** |
| ≥ 12 weeks | 14 | -5.14 (-6.81, -3.46) | 0.000 | 80.2 |  |
| < 12 weeks | 3 | -42.607 (-46.877 , -38.337) | 0.000 | 99.3 |  |
| **Matching** |  |  |  |  | **0.000** |
| Yes | 11 | -4.41 (-6.39, -2.42) | 0.000 | 73.1 |  |
| No | 6 | -19.288 (-21.800 , -16.775) | 0.000 | 99 |  |
| **Adjustment for baseline serum zinc** |  |  |  |  | **0.000** |
| Yes | 13 | -5.142 (-7.216 , -3.068) | 0.000 | 73.9 |  |
| No | 4 | -16.58 (-18.94, -14.22) | 0.000 | 99.4 |  |
| **Baseline serum zinc Status** |  |  |  |  | **0.000** |
| Deficient | 2 | -12.836 (-19.498 , -6.173) | 0.000 | 94.6 |  |
| Sufficient | 11 | -4.317 (-6.499 , -2.135) | 0.016 | 54.1 |  |
| Not Reported | 4 | -16.586 (-18.945 , -14.226) | 0.000 | 99.4 |  |
| **Study Quality** |  |  |  |  | **0.000** |
| Good | 9 | -4.31(-6.72 , -1.60) | 0.030 | 53.0 |  |
| Fair | 3 | -25.34 (-31.22 , -19.45) | 0.000 | 99.4 |  |
| Poor | 5 | -12.81 (-14.99 , -10.63) | 0.000 | 98.1 |  |

**Table 1.** Subgroup analysis for the effects of zinc supplementation on TC

**Table 2.** Subgroup analysis for the effects of zinc supplementation on HDL

| Group | Comparisons, n | WMD (95% CI) | P-value^1^ | I^2^ (%) | P-heterogeneity^2^ |
| --- | --- | --- | --- | --- | --- |
| **Total** | **16** | **3.762 (1.303 , 6.220)** | **0.000** | **96.7** |  |
| **Study Design** |  |  |  |  | **0.000** |
| Randomized | 3 | 9.00 (7.62, 10.37) | 0.000 | 94.3 |  |
| Randomized Blinded | 5 | 1.24 (0.71, 1.77) | 0.000 | 98.4 |  |
| Parallel Randomized Blinded | 8 | 1.07 (0.34, 1.81) | 0.000 | 87.5 |  |
| **Sex** |  |  |  |  | **0.000** |
| Male | 4 | 1.18 ( 0.63, 1.74) | 0.000 | 98.8 |  |
| Female | 2 | 0.00 ( -1.07, 1.07) | 1.000 | - |  |
| Both | 10 | 4.152 (3.394 , 4.910) | 0.000 | 93.7 |  |
| **Country** |  |  |  |  | **0.000** |
| Developed | 2 | 0.00 (-1.07, 1.07) | 1.000 | - |  |
| Developing | 14 | 2.214 (1.768 , 2.660) | 0.000 | 97.0 |  |
| **Age** |  |  |  |  | **0.000** |
| ≤60 years | 9 | 3.702 (2.884 , 4.520) | 0.000 | 95.3 |  |
| >60 years | 3 | 0.86 (-0.03, 1.76) | 0.015 | 76.1 |  |
| Not Reported | 4 | 1.43 (0.86, 1.99) | 0.000 | 98.8 |  |
| **Health Condition** |  |  |  |  | **0.000** |
| With micro albuminuria /nephropathy | 5 | 1.16 (0.64, 1.68) | 0.000 | 95.5 |  |
| Without micro albuminuria /nephropathy | 11 | 3.066 (2.398 , 3.733) | 0.000 | 97.1 |  |
| **Interventions Type** |  |  |  |  | **0.005** |
| Sulfate | 10 | 1.56 ( 1.09, 2.03) | 0.000 | 97.4 |  |
| Gluconate | 3 | 2.13 ( 0.78, 3.49) | 0.000 | 91.9 |  |
| Mixed | 3 | 3.557 (2.444 , 4.671) | 0.000 | 97 |  |
| **Control Type** |  |  |  |  | **0.002** |
| Placebo | 13 | 1.62 ( 1.18, 2.06) | 0.000 | 96.8 |  |
| Non-Placebo | 3 | 3.557 (2.444 , 4.671) | 0.000 | 94.2 |  |
| **Duration** |  |  |  |  | **0.000** |
| ≥ 12 weeks | 13 | 1.48 ( 1.05, 1.91) | 0.000 | 96.8 |  |
| < 12 weeks | 3 | 6.416 (4.982 , 7.851) | 0.000 | 94.2 |  |
| **Matching** |  |  |  |  | **0.000** |
| Yes | 10 | 1.32 ( 0.85, 1.78) | 0.000 | 93.4 |  |
| No | 6 | 3.921 (3.039 , 4.804) | 0.000 | 98.3 |  |
| **Adjustment for baseline serum zinc** |  |  |  |  | **0.000** |
| Yes | 13 | 1.196 (0.770 , 1.623) | 0.000 | 96 |  |
| No | 3 | 11.26 ( 9.69, 12.84) | 0.221 | 33.8 |  |
| **Baseline serum zinc Status** |  |  |  |  | **0.000** |
| Deficient | 2 | 3.988 (1.887 , 6.090) | 0.049 | 74.3 |  |
| Sufficient | 11 | 1.076 (0.641 , 1.512) | 0.000 | 96.6 |  |
| Not Reported | 3 | 11.269 (9.697 , 12.840) | 0.221 | 33.8 |  |
| **Study Quality** |  |  |  |  | **0.000** |
| Good | 9 | 1.11 ( -0.64 ,1.57 ) | 0.000 | 97.3 |  |
| Fair | 3 | 0.37 ( -1.22 ,1.95 ) | 0.154 | 46.6 |  |
| Poor | 4 | 6.90 (5.81 , 7.99) | 0.000 | 94.9 |  |

| Group | Comparisons, n | WMD (95% CI) | P-value^1^ | I^2^ (%) | P-heterogeneity^2^ |
| --- | --- | --- | --- | --- | --- |
| **Total** | **17** | **-6.185 (-9.349 , -3.021)** | **0.289** | **14** |  |
| **Study Design** |  |  |  |  | **0.678** |
| Randomized | 3 | -4.14 (-10.59 , 2.31) | 0.072 | 62.1 |  |
| Randomized Blinded | 5 | 0.33 (-13.98, 14.66) | 0.968 | 0.0 |  |
| Parallel Randomized Blinded | 9 | -5.57 (-7.80 , -3.34) | 0.151 | 33.4 |  |
| **Sex** |  |  |  |  | **0.494** |
| Male | 4 | -3.53 (-19.39, 12.32) | 0.992 | 0.0 |  |
| Female | 2 | -7.60 (-11.97, -3.23) | 0.851 | 0.0 |  |
| Both | 11 | -4.639 (-7.035 , -2.242) | 0.073 | 41.4 |  |
| **Country** |  |  |  |  | **0.238** |
| Developed | 2 | -7.60 (-11.97, -3.23) | 0.851 | 0.0 |  |
| Developing | 15 | -4.614 (-6.983 , -2.244) | 0.246 | 18.5 |  |
| **Age** |  |  |  |  | **0.026** |
| ≤60 years | 10 | -3.877 (-6.330 , -1.423) | 0.656 | 0 |  |
| >60 years | 3 | -7.61 (-11.85, -3.36) | 0.982 | 0 |  |
| Not Reported | 4 | -17.21 (-27.78, -6.64) | 0.218 | 32.4 |  |
| **Health Condition** |  |  |  |  | **0.775** |
| With micro albuminuria /nephropathy | 5 | -3.28 (-17.19, 10.62) | 0.816 | 0.0 |  |
| Without micro albuminuria /nephropathy | 12 | -5.340 (-7.446 , -3.234) | 0.109 | 35.2 |  |
| **Interventions Type** |  |  |  |  | **0.827** |
| Sulfate | 11 | -4.82 (-7.39, -2.25) | 0.121 | 34.7 |  |
| Gluconate | 3 | -6.84 (-21.54, 7.85) | 0.755 | 0.0 |  |
| Mixed | 3 | -6.146 (-9.798 , -2.494) | 0.310 | 14.7 |  |
| **Control Type** |  |  |  |  | **0.578** |
| Placebo | 14 | -4.88 (-7.41, -2.34) | 0.251 | 18.5 |  |
| Non-Placebo | 3 | -6.146 (-9.798 , -2.494) | 0.310 | 14.7 |  |
| **Duration** |  |  |  |  | **0.001** |
| ≥ 12 weeks | 14 | -4.56 (-6.69, -2.44) | 0.981 | 0.0 |  |
| < 12 weeks | 3 | -22.741 (-33.158 , -12.324) | 0.262 | 25.4 |  |
| **Matching** |  |  |  |  | **0.102** |
| Yes | 11 | -4.00 (-6.59, -1.41) | 0.992 | 0.0 |  |
| No | 6 | -7.650 (-12.043 , -3.257) | 0.019 | 62.9 |  |
| **Adjustment for baseline serum zinc** |  |  |  |  | **0.766** |
| Yes | 13 | -5.700 (-9.089 , -2.312) | 0.986 | 0.0 |  |
| No | 4 | -5.04 (-7.68, -2.40) | 0.002 | 76.9 |  |
| **Baseline serum zinc Status** |  |  |  |  | **0.368** |
| Deficient | 2 | -1.693 (-8.310 , 4.923) | 0.784 | 0 |  |
| Sufficient | 11 | -7.125 (-11.071 , -3.180) | 0.997 | 0 |  |
| Not Reported | 4 | -5.047 (-7.687 , -2.407) | 0.002 | 79.6 |  |
| **Study Quality** |  |  |  |  | **0.032** |
| Good | 9 | -7.12 (-11.15, -3.08) | 0.999 | 0.0 |  |
| Fair | 3 | -19 (-30.93 , -7.08) | 0.077 | 61 |  |
| Poor | 5 | -4.01 (-6.94 , -1.53) | 0.217 | 14 |  |

**Table 3.** Subgroup analysis for the effects of zinc supplementation on LDL

| Group | Comparisons, n | WMD (95% CI) | P-value^1^ | I^2^ (%) | P-heterogeneity^2^ |
| --- | --- | --- | --- | --- | --- |
| **Total** | **17** | **-13.084 ( -21.826 , -4.342)** | **0.000** | **88.4** |  |
| **Study Design** |  |  |  |  | **0.000** |
| Randomized | 3 | -38.480 ( -46.703 , -30.258) | 0.000 | 88.1 |  |
| Randomized Blinded | 5 | -5.891 (-14.773 , 2.990) | 0.016 | 67 |  |
| Parallel Randomized Blinded | 9 | -4.745 ( -7.536 , -1.953) | 0.000 | 84.4 |  |
| **Sex** |  |  |  |  | **0.000** |
| Male | 4 | -6.296 (-16.291 , 3.698) | 0.012 | 72.5 |  |
| Female | 2 | 0.162 (-4.570 , 4.895) | 0.000 | 92.5 |  |
| Both | 11 | -11.836 (-14.981 , -8.692) | 0.000 | 89.7 |  |
| **Country** |  |  |  |  | **0.000** |
| Developed | 2 | 0.162 (-4.570 , 4.895) | 0.000 | 92.5 |  |
| Developing | 15 | -11.337 (-14.337 , -8.338) | 0.000 | 87.1 |  |
| **Age** |  |  |  |  | **0.000** |
| ≤60 years | 10 | -11.440 (-14.647 , -8.233) | 0.000 | 89.3 |  |
| >60 years | 3 | -0.117 (-4.620 , 4.387) | 0.001 | 85.2 |  |
| Not Reported | 4 | -14.542 (-24.932 , -4.152) | 0.000 | 87.1 |  |
| **Health Condition** |  |  |  |  | **0.183** |
| With micro albuminuria /nephropathy | 5 | -13.819 (-22.691 , -4.948) | 0.000 | 90.4 |  |
| Without micro albuminuria /nephropathy | 12 | -7.528 (-10.172 , -4.884) | 0.000 | 88.4 |  |
| **Interventions Type** |  |  |  |  | **0.009** |
| Sulfate | 11 | -5.757 (-8.683 , -2.8) | 0.000 | 88.7 |  |
| Gluconate | 3 | -15.508 (-24.360 , -6.6) | 0.020 | 74.6 |  |
| Mixed | 3 | -14.575 (-20.749 , -8.4) | 0.000 | 93.9 |  |
| **Control Type** |  |  |  |  | **0.023** |
| Placebo | 14 | -6.718 (-9.496 , -3.939) | 0.000 | 87 |  |
| Non-Placebo | 3 | -14.575 (-20.749 , -8.401) | 0.000 | 93.9 |  |
| **Duration** |  |  |  |  | **0.000** |
| ≥ 12 weeks | 14 | -4.424 (-7.139 , -1.709) | 0.000 | 82.9 |  |
| < 12 weeks | 3 | -32.403 (-39.450 , -25.357) | 0.008 | 79.8 |  |
| **Matching** |  |  |  |  | **0.701** |
| Yes | 11 | -7.647 (-10.881 , -4.413) | 0.000 | 83.2 |  |
| No | 6 | -8.667 (-12.744 , -4.590) | 0.000 | 93.6 |  |
| **Adjustment for baseline serum zinc** |  |  |  |  | **0.005** |
| Yes | 13 | -4.573 (-8.072 , -1.075) | 0.000 | 74.5 |  |
| No | 4 | -11.867 (-15.541 , -8.192) | 0.000 | 96.4 |  |
| **Baseline serum zinc Status** |  |  |  |  | **0.014** |
| Deficient | 2 | 1.41 (-14.973 , 17.798) | 0.974 | 0 |  |
| Sufficient | 11 | -4.85 (-8.440 , -1.279) | 0.000 | 78.5 |  |
| Not Reported | 4 | -11.87 (-15.54 , -8.19) | 0.000 | 96.4 |  |
| **Study Quality** |  |  |  |  | **0.001** |
| Good | 9 | -4.44 (-6.26 , 1.42) | 0.000 | 74.2 |  |
| Fair | 3 | -12.40 ( -26.67 , 1.87) | 0.000 | 88.6 |  |
| Poor | 5 | -12.29 (-15.75 , -8.83) | 0.000 | 94.7 |  |

**Table 4.** Subgroup analysis for the effects of zinc supplementation on TG

LDL: Low Density Lipoprotein; HDL: High Density Lipoprotein; No: Number; TC: Total Cholesterol; TG:
Triglyceride; WMD: Weighted Mean Difference

^1^P for within subgroup heterogeneity

^2^P for between subgroup heterogeneity

**Supplementary figure legends**

**Supplementary Fig 1.** Funnel plot analysis of Publication Bias.


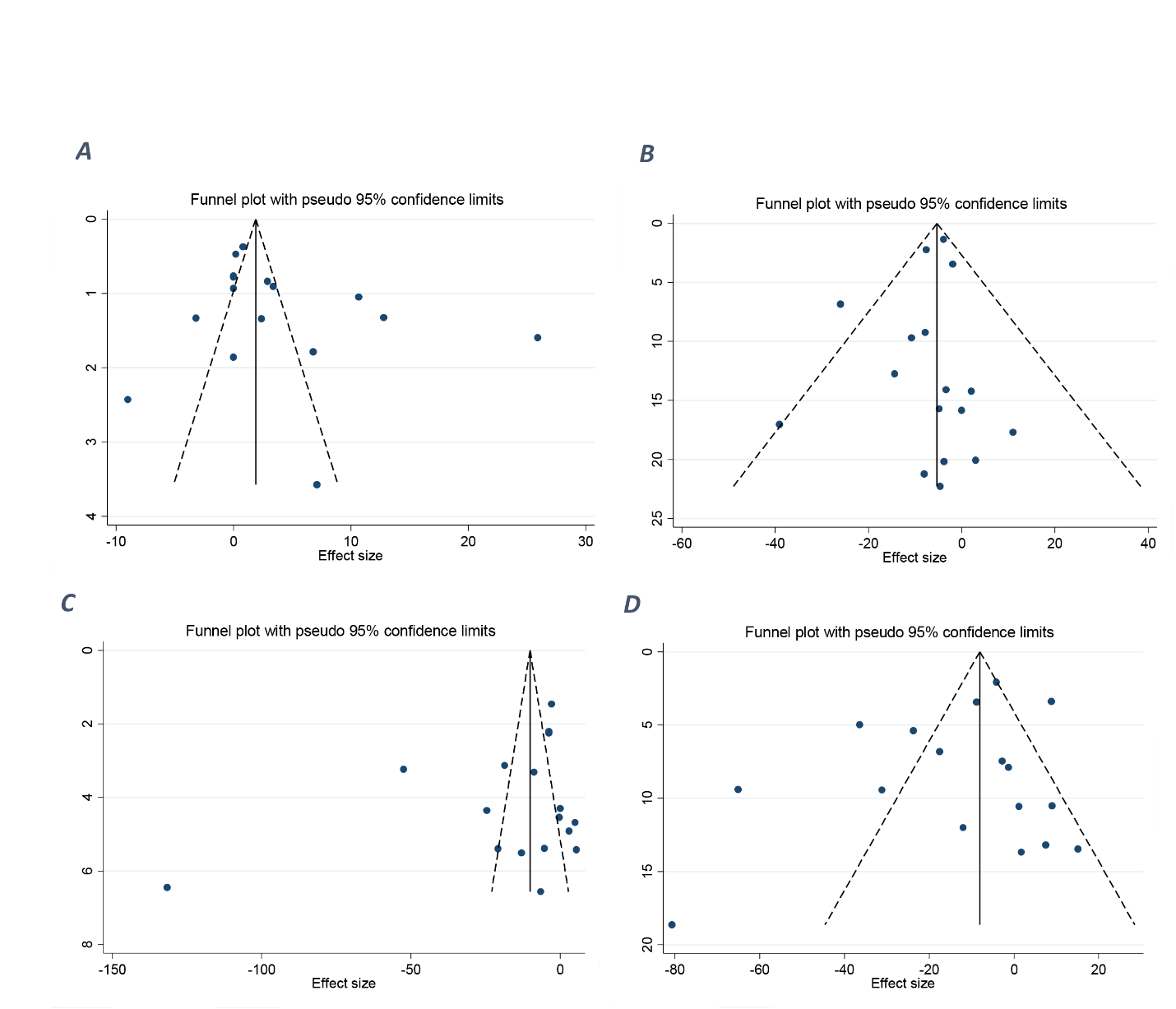


^A^: HDL; ^B^: LDL; ^C^: TC; ^D^: TG
